# Lymphocyte homeostasis is maintained in perinatally HIV-infected patients after three decades of life

**DOI:** 10.1186/s12979-019-0166-7

**Published:** 2019-10-13

**Authors:** S. Paghera, E. Quiros-Roldan, A. Sottini, M. Properzi, F. Castelli, L. Imberti

**Affiliations:** 1 Centro di Ricerca Emato-oncologica AIL (CREA), Diagnostic Department, ASST Spedali Civili, Brescia, Italy; 2 Department of Infectious and Tropical Diseases, University of Brescia and ASST Spedali Civili, Brescia, Italy

**Keywords:** Perinatal HIV infection, Immunosenescence, T-cell receptor repertoire, Telomere length, Thymic and bone marrow output

## Abstract

**Background:**

While immunosenescence, defined as reduced production of new lymphocytes, restriction of T-cell receptor repertoire and telomeres shortening, has been extensively evaluated in HIV-infected children and adults, no data about these parameters are available in perinatally-infected patients with very long-lasting HIV infection.

**Methods:**

We compared thymic and bone marrow output, telomere length (measured by Real-Time PCR) and T-cell receptor repertoire (determined by spectratyping) of 21 perinatally HIV-infected subjects (with a median of 27 years of infection) with those of 19 age-matched non-perinatally HIV-infected patients and 40 healthy controls. All patients received a combined antiretroviral therapy.

**Results:**

While thymic and bone marrow output were not different among the analyzed groups, telomere length in peripheral blood cells and T-cell receptor diversity were significantly lower in HIV-perinatally and non-perinatally infected individuals compared to healthy controls.

**Conclusions:**

In HIV-infected subjects, a normal thymic output together with a reduced telomere length and a restricted T-cell receptor repertoire could be explained by the shift of newly produced cells into memory subsets. This phenomenon may allow to control viral infection and maintain peripheral homeostasis.

## Introduction

After the introduction of combined antiretroviral therapy (cART), the rate of vertical HIV transmission drastically dropped to 1–2%, or even lower, in the United States and Western Europe [1]. To date, an increasing proportion of people living with HIV is growing older, with a not negligible percentage of perinatally HIV-infected youths (pHIVy) gradually becoming adults. pHIVy present a higher risk of treatment failure, progression to AIDS [2], co-morbidities [3] and increased mortality [4], primarily due to immune system alterations (as chronic immune activation and exhaustion) [5, 6] that remain evident even after therapy-induced virologic suppression.

Peripheral T, B and NK lymphocytes of HIV-infected children and adults show features that have been considered as hallmarks of premature aging process. Immunosenescence includes both a reduced production of new T and B cells and an increased proliferation of the existing T cells, leading to a restriction of T-cell receptor (TR) repertoire and to a more rapid telomere length (TL) shortening [7, 8]. Therefore, a typical element of accelerated aging is a reduced thymic output, indicated by a decreased production of recent thymic emigrants, containing TR excision circles (TRECs) [9–13]. In adults, long-term cART only partially reverses HIV-mediated loss of CD4^+^ T cells and does not completely renovate TR repertoire diversity [14–18]. On the contrary, in HIV-positive children, cART induces an early sustained increase in naïve CD4^+^ T cells, likely reflecting a greater thymic activity compared with adults [19–21], and it is also responsible of the increased TR repertoire diversity observed within several months of therapy in these patients [22]. Therefore, the role of thymic activity in the immune recovery of severely lymphopenic patients is gradually emerging [23].

Moreover, remaining one of the main characteristics of immunosenescence, TL shortening reported in HIV-infected children [24] and adults [25–27] has also been associated with nucleoside reverse transcriptase inhibitors-based regimens, which are proven to inhibit telomerase activity in-vitro [28]. Thus, some authors suggested that HIV-related immunosenescence can also be ascribed to cART adverse effects.

Other peculiar immunological age-linked features of HIV-infected patients are the phenotypic and functional alterations in B cells and the defects in antibody production, observed both in children with perinatal HIV infection [29–31] and adults [32–36]. Similarly to T cells, memory B cells deficits persists together with an increase in other cell subsets, even after cART initiation [32, 37, 38].

All previous reported studies included perinatally-infected children and adults with sexually or intravenous-acquired HIV infection. The ones performed in adolescent and young survivors of perinatal HIV infection are incomplete and limited as broad age ranges were used; moreover, data were only compared with healthy controls (HC) [39, 40], but not with age-matched non perinatally-HIV-infected youths (npHIVy). Therefore, our aim was to compare thymic and bone marrow output, TR repertoire and TL of pHIVy, with almost three decades of infection, to those of age-matched npHIVy (both on stable cART and long-term virologically suppressed) and HC.

## Methods

### Patients

This is a single center, cross-sectional, non-interventional study including HIV-infected patients followed at the Department of Infectious and Tropical Diseases of ASST Spedali Civili of Brescia. Twenty-one pHIVy and 19 age-matched npHIVy were enrolled between March and December 2018. npHIVy were included only if HIV infection duration was > 12 months, in order to exclude acute infection. Other exclusion criteria were an ongoing hepatitis C virus treatment or having any serious concomitant disease/ongoing infection.

All HIV-infected patients were treated with cART according to current guidelines for HIV treatment and all presented HIV-RNA < 200 copies/ml.

Results were compared with those of 40 age-matched HC, whose anonymized biological samples were obtained as remained/discarded blood after routine analysis at the Laboratory of the ASST Spedali Civili of Brescia.

### Quantification of thymic and bone marrow output

The number of TRECs and K-deleting recombination excision circles (KRECs) was simultaneously quantified by duplex quantitative Real-Time PCR (qPCR), using DNA obtained from peripheral blood mononuclear cells, as previously reported [41]. Results were expressed as copies/ml of blood.

### TL measurement

Relative TL was measured by monochrome multiplex PCR, as previously reported [42], with minor modifications. DNA was extracted using QIAamp DNA Blood Mini Kit (Qiagen GmbH, Hilden, Germany), as recommended [43], from peripheral blood mononuclear cells obtained by Ficoll separation; its integrity was guaranteed by visualization on agarose gels, as suggested [44], while its quantity and quality were assessed on Nanodrop 2000 spectrophotometer. DNA stocks were diluted at 10 ng/μl just prior to set up the runs. Amplification was performed using commercial HRM master mix (MeltDoctor™ HRM Master Mix, Applied Biosystems, Foster City, CA) to avoid repeated pipetting of single reagents [45]. For all experiments the same lot of each reagent was used. All experimental plates included a reference DNA (standard curve generated by 1:2 serial dilutions, from 21 to 0.16 ng/well), obtained by mixing DNA prepared from peripheral blood mononuclear cells of 8 HC. Experimental samples, homemade internal controls (consisting of same batches of DNA extracted from cord blood mononuclear cells, containing long telomeres, and DNA extracted from Jurkatt cell line, containing short telomeres) and a negative control (no DNA template) were evaluated in triplicate in each run.

The T/S ratio for an experimental DNA sample is T (the number of nanograms of the reference DNA that matches the experimental sample for copy number of the telomere template) divided by S (the number of nanograms of the reference DNA that matches the experimental sample for copy number of beta-globin single copy gene).

Mean qPCR efficiency was 115% for telomere and 105% for beta-globin gene; slopes of the standard curves were − 3.05 and − 3.22, while the coefficient of regression (r^2^) was 0.98 and 0.99 for telomere and beta-globin gene, respectively. Values were not accepted if the 3 replicates had a CV > 15% [46]. The intra-assay and inter-assay CV, calculated on the T/S ratio, were 6.7% (as in Aviv et al.) [47] and 12%, respectively.

### TR repertoire analysis

The analysis of TR beta variable (TRBV) subgroups by complementarity determining region 3 (CDR3) spectratyping was performed, as previously described [48], using 500 ng of total RNA extracted from at least 2 × 10^6^ cells. The first strand of cDNA, obtained with random examers, was amplified by multiplex PCRs, allowing the detection of 23 TRBV subgroups [49]. The distribution of fragment lengths, number of detectable peaks per TRBV subgroup and area under the curve of PCR products were calculated with GeneMapper 5 software (Applied Biosystems, Foster city, CA), as previously reported [50]. We used the nomenclature of TRBV subgroups according to IMGT (http://www.imgt.org/).

### Statistical analysis

GraphPad Prism version 5.1 (GraphPad Software, San Diego, CA) was used for statistical analysis. Comparisons among medians of the quantitative variables were performed by non parametric Kruskal-Wallis H Test, since the variables were not normally distributed. When the interaction was significant, post-hoc Dunn’s test corrected *P*-values were calculated. In case the significant effect included only two levels, its *P*-value was reported. The Pearson’s correlation was calculated to verify the possible association with age and gender of the variables used. In Table 1, P-value calculations were obtained by Fisher exact test for categorical variables and unpaired t test for continuous variables. *P*-values ≤0.05 were considered significant.

**Table 1:** Characteristics of included HIV^+^ subjects and controls

	pHIVy patients (# = 21)	npHIVy patients (# = 19)^a^	HC (# = 40)
Age (years); median (IQR)	27 (24–29)	27 (24–29)	28 (24–31)
Males; patients (%)	7 (33)	12 (63)	19 (47.5)
CD4/μl; median (IQR)	803 (526–1052)	818 (688–1024)	851 (643–1099)
CD4, %; median (IQR)	33.8 (28.7–38.8)	38.1 (31.9–42)	42.9 (39.7–49.4)
CD8/μl; median (IQR)	850 (713–1131)	728 (631–812)	415 (344–571)
CD8, %; median (IQR)	42.8 (32.7–51)	32.3 (28–40.3)	22.2 (22.0–22.6)
CD4/CD8 ratio; median (IQR)	0.8 (0.6–1.2)	1.2 (0.8–1.4)	2.0 (2.0–2.5)
Years with HIV; median (IQR)	27* (0)	4* (3–5.5)	NA
HIV viral load < 37 copies/ml; patients	20	19	NA
Hepatitis B surface antigen positive; patients (%)	1 (4.76)	1 (5.26)	NA
Hepatitis C virus-antibody positive; patients (%)^b^	5 (23.81)	1 (5.26)	NA
Cytomegalovirus IgG positive; patients (%)	13 (61.90)	7 (36.84)	NA
Toxoplasma gondii IgG positive; patients (%)	4 (19.04)	2 (10.52)	NA
Treatment prescribed at enrolment:			
PI + 2 NRTI; patients (%)	4 (19)	5 (26)	NA
NNRTI + 2 NRT; patients (%)	3 (14)	8 (42)	NA
INI + 2 NRTI; patients (%)	6 (29)	6 (32)	NA
INI + PI; patients (%)	7 (33)	0	NA
INI + NNRTI; patients (%)	1 (5)	0	NA

*HC* healthy controls, *IQR* interquartile range, *NA* not applicable, *PI* protease inhibitor, *NRTI* nucleoside reverse transcriptase inhibitor, *NNRTI* non nucleoside reverse transcriptase inhibitor, *INI* integrase inhibitor, *#* number

*P < 0.001 (P-value calculation was done by Fisher exact test for categorial variables and unpaired t test for continuous variables)

^a^ 9 patients were heterosexual, 8 homosexual and 2 bisexual. ^b^All 6 patients were Sustained Virological Responsers (SVR) to previous anti-HCV therapy with Directly Acting Antivirals (DAA)

### Ethics

The study was approved by the ethics committee of Brescia on March 2018 (NP 3061) and was conducted according to the principles expressed in the Declaration of Helsinki and its later amendments. Written informed consent was obtained from all patients.

## Results

### Study population

In our study we included 21 pHIVy with a median age of 27 years [Interquartile range (IQR): 24–29 years] and two age-matched control groups: 19 npHIVy, with a median duration of HIV infection of 4 years (IQR: 3–5.5 years), and 40 HC. CD4^+^ cell count was similar among the three groups considered (median 803/μl, 818/μl and 851/μl, respectively). All HIV-infected participants were on stable cART and the vast majority was long-term virologically suppressed. Therefore, HIV viral load was constantly < 37 copies/ml in all (except one) pHIVy throughout the follow-up at our Unit, started from the age of 18. Similarly, all npHIVy were virologically suppressed at 6 months after first cART-initiation. The characteristics of the study population are listed in Table 1.

### Thymic and bone marrow output and TL

**Fig. 1:**
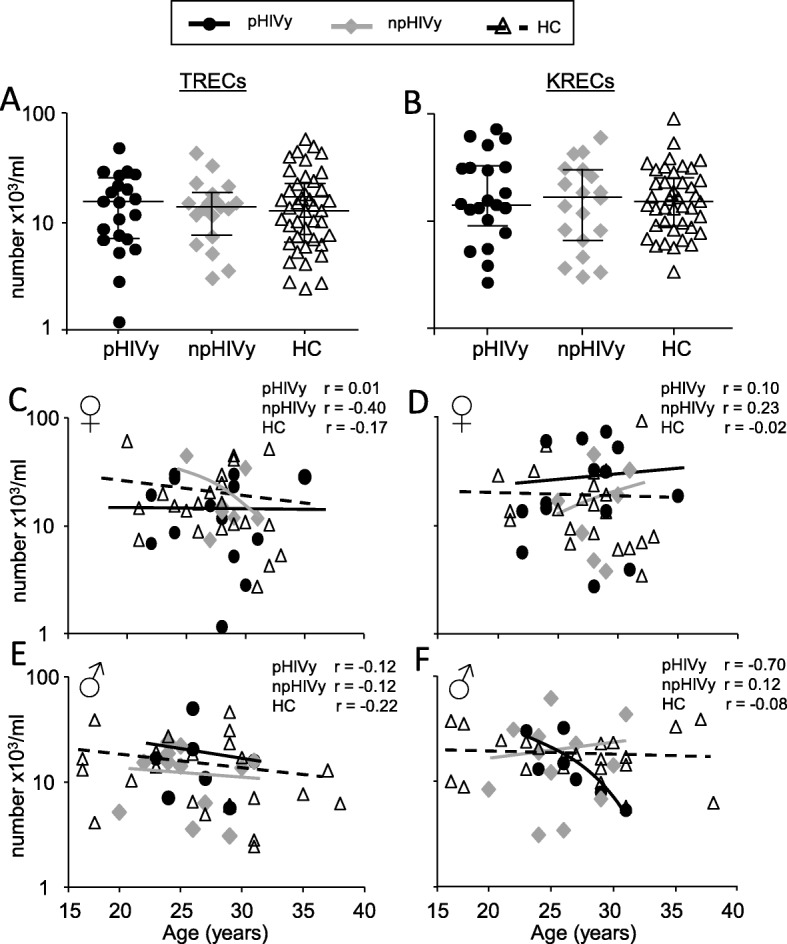
TRECs (**a**) and KRECs (**b**) in pHIVy, npHIVy and HC. Correlation between TRECs (**c**, **e**), KRECs (**d**, **f**) and age according to gender. Legend: pHIVy: perinatally HIV-infected youths, npHIVy: non-perinatally HIV-infected youths, HC: healthy controls. Bars indicate median and IQR values

The release of new T and B lymphocytes from the production sites, measured by TRECs and KRECs quantification, was not statistically different in HIV-infected individuals, neither if they acquired the virus in the perinatal period nor as young adults (Fig. 1a, b). Moreover, TRECs and KRECs levels were comparable to those of age-matched HC and no differences were observed depending on age and gender (Fig. 1c, d, e, f and Additional file 1: Table S1).

**Fig. 2:**
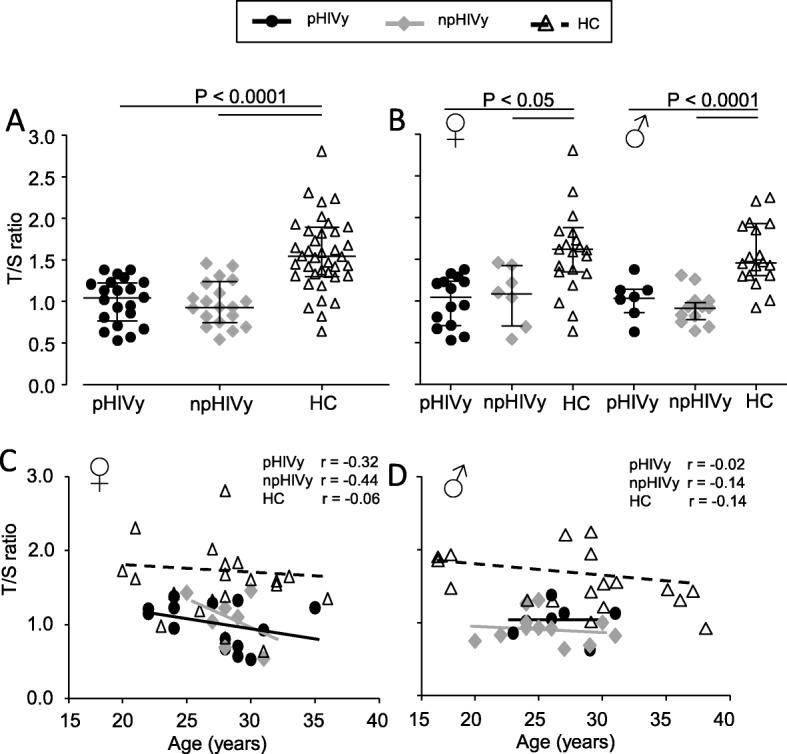
Telomere length (as T/S ratio) in pHIVy, npHIVy and HC (**a**). Telomere length according to gender (**b**). Correlation between telomere length and age according to gender (**c**, **d**). Legend: pHIVy: perinatally HIV-infected youths, npHIVy: non-perinatally HIV-infected youths, HC: healthy controls. Bars indicate median and IQR values (**a** and **b**). Black and gray continuous lines and the dotted line indicate the correlation between T/S and age (**c** and **d**)

Median TL value, expressed as T/S ratio, in peripheral blood cells was significantly lower in pHIVy and npHIVy, than in HC (Fig. 2a). These differences remained even dividing the two groups of HIV-positive patients according to gender (Fig. 2b). No correlation between TLs, age and gender was found (Fig. 2c, d). Finally, there was no association between TLs and the ongoing antiretroviral regimen (data not shown).

As expected, we observed a direct relationship between the number of cells containing TRECs and CD4^+^ cell count, as well as CD8^+^ and CD4/CD8 ratio. Moreover, we found a strong positive correlation only for CD4^+^ lymphocytes in pHIVy (r = 0.75; *P* < 0.0001; Additional file 2: Figure S1). Similarly, a strong significant correlation was observed between the extent of TL and the number of CD4^+^ cells in the same group of patients (r = 0.75; *P* = 0.0001). Similar results were obtained using CD4^+^ cells percentage with both TRECs and TL (data not shown). No significant correlations were found in HC group.

### TR repertoire

The diversity of TR repertoire was analyzed by medians of CDR3 spectratyping analysis. The most utilized TRBV subgroups were TRBV2, TRBV4, TRBV6, TRBV11 and TRBV12 in the three groups of analyzed subjects. The usage of TRBV subgroups was very similar in the two groups of HIV-infected individuals and was superimposable to that of HC. The only exception was TRBV7 which, despite being very similar in the two groups of HIV-infected individuals, was significantly more used only in npHIVy than in HC (see Additional file 3: Figure S2).

**Fig. 3:**
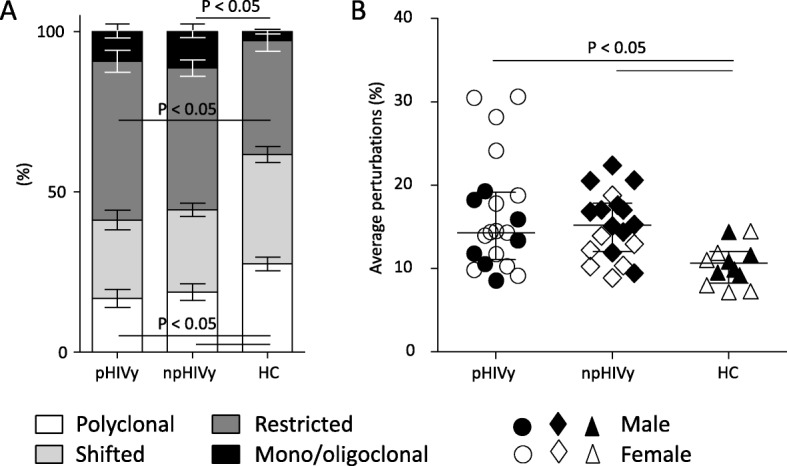
Percentage of polyclonally distributed, shifted, restricted, and mono/oligoclonally expanded TR repertoire (**a**) and average percentages of TRBV perturbations (**b**).Legend: The complexity of individual TRBV CDR3 size distributions was determined by counting the number of distinct peaks within each TRBV subgroup to classify it into 4 categories: normal (≥7 peaks, Gaussian distribution), shifted (≥7 peaks, deviation from Gaussian distribution), restricted (< 7 peaks prominent deviation from Gaussian distribution), and mono/oligoclonal (one or two dominant peaks). For each individual, the proportions of TRBV subgroups with a CDR3 distribution belonging to each one of these categories were calculated. The reported significance was obtained comparing the within-patient proportions of pooled shifted, restricted and mono/oligoclonal vs. normal polyclonal TRBV subgroups in patients and HC. Average percentages of TRBV perturbations; each dot represents the global average perturbation of the TRBV repertoire in each subject (B). Displayed results were calculated in perinatally HIV-infected youths (pHIVy), in non-perinatally HIV-infected youths (npHIVy) and healthy controls (HC). Bars indicate median and IQR values

**Fig. 4:**
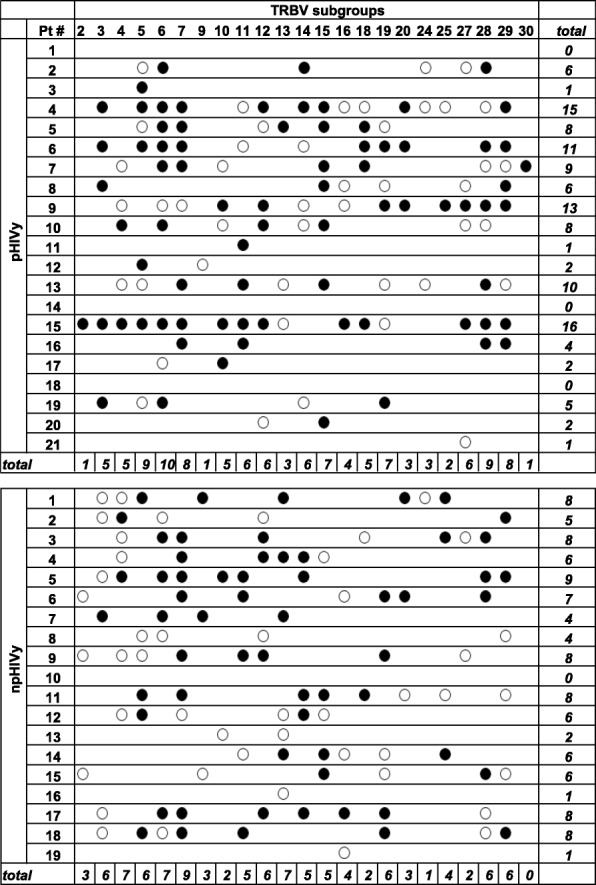
Distribution of CDR3 perturbations calculated at single-TRBV subgroup and single-patient level. Legend: Perturbations distribution was calculated with the generalized Hamming distance method [50], by “subtracting” from the CDR3 length distribution of each TRBV of a single patient the average CDR3 length distribution obtained by analyzing a “reference group” of 12 HC. Black and white dots represent the TRBV subgroups whose perturbations are higher than the mean + 3SD and mean + 2SD, respectively, of the value seen in the corresponding TRBV subgroup in HC. The sum of these over-perturbed TRBV subgroups in each individual is indicated in the right column. The number of patients in whom each TRBV subgroup is perturbed is indicated at the bottom. HC: healthy controls; npHIVy: non-perinatally HIV-infected youths; pHIVy: perinatally HIV-infected youths; pt. #: patient number; TRBV: T-cell receptor variable beta

The percentage of TRBV subgroups with normal (polyclonal) profiles was significantly lower in samples obtained from the two groups of HIV-infected patients in comparison to HC (16.8 and 18.8% vs 27.5%, Fig. 3a). The proportion of TRBV subgroups with shifted profiles was not different, that with restricted TRBV profiles was significantly higher in pHIVy than in HC (49.5% vs 35.5%), but mono/oligoclonal TRBV cells were higher in npHIVy than in HC (11.4% vs 2.9%). Although median percentages of TRBV perturbations were significantly higher in both pHIVy (14.4%) and npHIVy (15.1%) than in HC (10.4%), the most globally restricted repertoires were those of four pHIVy, displaying perturbations from 24.1 to 30.6% (Fig. 3b). Accordingly, if TRBV perturbations were evaluated both at single-TRBV subgroup and single-patient level, and by referring the data to those of HC, the highest number of TRBV restrictions was detected in the same subjects (#4, #6, #9 and #15; Fig. 4). Two of them were the only patients of our study with current CD4^+^ cells < 100/μl (61/μl for patient #6 and 85/μl for patient #15; CD4/CD8 ratio was 0.1 in both individuals), despite being aviremic. Patient #4 suffered from severe chronic obstructive pulmonary disease and despite having 526 CD4^+^ cells/μl, his CD4/CD8 ratio was 0.3. Last patient (#9), who had remained virologically suppressed in the last 6 years, showed 893 CD4^+^ cells/μl with a CD4/CD8 ratio of 0.6. None of the 4 patients with the highest number of TRBV restrictions was hepatitis C virus-antibody positive. In the group of npHIVy, patient #5 had the most restricted TR repertoire, with 9 perturbed TRBV subgroups. However, TR repertoires of pHIVy were the most heterogeneous, as this group included the highest number of individuals with less perturbed profiles. In fact, patients #1, #14, #18 showed no TRBV perturbations, patients #3, #11, #21 had only one perturbed TRBV subgroup, while for patients #12, #17, #20 two perturbed TRBV subgroups were reported. By contrast, in npHIVy, only patient #10 did not present any perturbed TRBVs, patients #16, #19 had one perturbed TRBV subgroup, while patient #13 showed two perturbed TRBV subgroups. In pHIVy, a strong negative correlation was found between the percentage of TRBV perturbations and CD4/CD8 ratio (r = − 0.81; *P* < 0.0001), while only a moderate correlation was observed with CD4^+^ cell count (r = − 0.63; *P* < 0.05; Additional file 4: Figure S3). The correlation was stronger using CD4^+^ cells percentage, instead of their absolute count (P < 0.0001; data not shown). A positive, although not significant, association was detected between the percentage of TRBV perturbations and the number of CD8^+^ cells.

## Discussion

Our results show that pHIVy and npHIVy, despite a very different duration of HIV infection, have comparable numbers of TRECs and KRECs in peripheral blood, which are also similar to those of HC. This comparable thymic and bone marrow output suggests a normal ongoing lymphopoiesis in all individuals included in the study. By contrast, the extent of telomere shortening and TRBV subgroup restriction were analogous between pHIVy and npHIVy, but significantly different compared with HC.

Most studies, both longitudinal and cross-sectional regarding immunosenescence in HIV infection, included acutely or chronically HIV-infected children or adults, whereas only few were performed in long-living, perinatally-infected patients [21]. The most recent one reported a low number of CD34^+^ hematopoietic progenitors and mature lymphocytes in young adults (HIV-infected perinatally or during childhood, on average 3 years after birth) [51]. Contrary to what has been observed in our cohort, a high proportion of these patients presented an active viral replication (33% vs 2.5%). Therefore, Authors indicated the uncontrolled viral load as a main cause of disease progression and elevated immune activation, resulting in a premature immune aging profile. The non-compliance of young HIV-infected patients with antiretroviral therapy represents a rather frequent and particularly sensitive issue [52]; thus, it is mandatory to enforce treatment adherence in teenagers and young adults, in order to prevent CD34^+^ hematopoietic progenitors and mature lymphocytes decrease. However, there are alternative reasons behind immunosenescence in HIV-patients with good control of viral replication, including low naïve CD4^+^ cells, increased T-cell death, immune hyperactivation, altered ratio of regulatory T and Th17 cells, tissue fibrosis and older age [53–55]. The release of new T cells from the production site seems to be the major driver of immune recovery during cART, being particularly relevant for patients who start their therapy with CD4^+^ T cells < 200/μl [23]. Accordingly, thymic output has been linked to HIV progression and CD4^+^ levels maintenance [56]. Several evidences show that immune reconstitution of T-cell compartments following cART clearly differs between children and adults. An early thymic output and a peripheral naïve T-cell repopulation may appear soon after cART initiation in children, while an expansion of memory T cells predominates in adults, in whom naïve CD4^+^ T cells are typically found only after several months of therapy [57]. A previous study regarding cART-treated pHIVy, with a shorter duration of HIV infection compared to our study (17 vs 27 years), found a more robust thymopoiesis in this population compared to HC of similar age [21]. This supranormal thymic output was attributed to a “thymic rebound”. However, TREC content is expected to normalize during time, as only few TREC^+^ T cells should be enough to replenish the virtually empty T-cell compartment. Accordingly, in bone marrow transplanted patients, the “lymphocyte rebound” is followed by a progressive decreased production of new T and B lymphocytes, starting about 2 years after transplantation [41]. Therefore, we favor the previously proposed hypothesis [12], suggesting that in adolescents and young adults with HIV infection the persistence of normal, good levels of thymic activity may potentially compensate for the deleterious effects of current and past HIV replication.

An accelerated TL shortening after HIV infection has also been described, but whether this event could lead to a premature senescence of immune cells remains unsettled, as several studies have shown inconsistent results in both untreated and cART-treated children [24, 58] and adults [25–27]. Here, we report a similar TL shortening in pHIVy and npHIVy, but higher compared with HC; moreover, we did not find neither gender-related differences nor association with any cART regimen (data not shown), as previously reported [28, 59].

It would be apparently difficult to conciliate the finding of a normal thymic output with an increased TL shortening. This apparent discrepancy could be explained by a continuous shift of the naïve pool into memory subsets that are under constant proliferation in the attempt to control viral infection and maintain peripheral homeostasis. Although we do not have the flow cytometric evidence of this shift, a recent study showed that in mice without infections to activate naïve T cells, there is a continuous influx of naïve T cells entering the pool of memory T cells (up to 10% per week) [60]. Thus, humans, who are continually exposed to pathogens, are likely to continually recruit new naïve T cells into the memory pool.

We also observed a reduction of TR repertoire diversity in all HIV-infected patients, supporting our hypothesis of a compensatory expansion of the existing T-cell population. Restricted TR repertoires are common features of HIV infection [16, 17, 61], although this status is highly dynamic with frequent reshaping [62], and, after cART, TR diversity requires years to return to normal ranges. Our detailed analysis of TRB CDR3 revealed that both groups of HIV-infected young adults have higher TRBV average perturbations in comparison with HC, but also that npHIVy showed higher percentage of oligo/monoclonal expanded TRBV subgroups, despite a shorter infection time and an efficient cART. One possible explanation is that pHIVy, despite the shift from naïve to memory cell phenotype, have a more remarkable thymic reserve, which may support some degree of TR repertoire normalization. We found a direct relationship between the number of cells containing TRECs and CD4^+^, indicating that a higher production of TRECs corresponds to a higher number of CD4^+^ lymphocytes in the periphery of pHIVy. Similarly, TRBV average perturbations negatively correlated with CD4+ absolute number and (in particular) percentage in pHIVy, while no significant correlation was found with CD8^+^. These findings suggest that CD4^+^ cell production depends on thymic output in this group of patients, while CD8^+^ cells might be the result of peripheral expansion. In addition, the positive correlation between the amount of CD4^+^ and T/S ratio indicates that, in these patients, CD4^+^ are, on average, new T cells which had undergone fewer cell divisions, resulting in telomere preservation. These results suggest that TRBV repertoire perturbations could be restricted to the antigen-experienced population of CD8^+^ cells, whereas perturbations may occur in naïve as well as in memory cells for CD4^+^. Since the same findings were observed in naïve younger (13–18 years old) HIV-positive adolescents (with much shorter HIV infection duration) [63], we can suppose that prolonged efficient cART, initiated early in life, could perpetuate this condition, despite a long-lasting HIV infection. It remains to be determined whether TR repertoire diversity loss in both groups of infected young adults is caused by CD8^+^ T-cell compartment expansion (which may directly reflect the increased prevalence of cytomegalovirus among HIV-infected individuals, as previously proposed) [64]. It could be also interesting to see whether the aberrant clonal/oligoclonal expansions observed in TR repertoires of npHIVy remain and play a role in chronic inflammation, contributing to long-term morbidity, even when viral load is controlled.

Little is known about B-lymphocyte recovery among early and chronically HIV-infected patients. Recently, higher KRECs levels were observed in HIV-infected children compared with uninfected healthy controls during the first 3 months of life [65]. This difference was explained by a greater differentiation from naïve to memory B cells and by higher rates of cell turnover, which lead to a compensatory increase in new B cells from the bone marrow. This probably represents a real “bone marrow rebound” occurring in a very peculiar situation, as bone marrow output reaches the highest levels soon after birth; however, HIV-infected patients can unlikely support such a high B-cell production. Comparable levels of KRECs in all groups analyzed in this study just confirm that HIV targets different subsets of B cells (such as plasmablasts, activated, resting memory and atypical or exhausted memory B cells), while the virus seems to have little or no influence on the ongoing release from bone marrow in young adults.

Although the almost three decades of age of our pHIVy and the inclusion of two control groups (npHIVy and HC) added value to this work compared to others, our analysis is limited by the relatively low number of subjects included and by its cross-sectional nature. We are also aware that several methods are available for TL quantification, such as terminal restriction fragment analysis, absolute quantification, various FISH techniques, and, in particular, the Telomere Shortest Length Assay (TeSLA), which additionally gives information on the shortest telomeres [66, 67]. Among them, we chose qPCR because of our expertise with this technique and because it is the most cost-effective and suitable method [43, 68], provided that certain conditions are respected, including a careful methodological analysis of each step of this process [43]. Finally, in our study all markers were evaluated analyzing peripheral blood cells pool; therefore, further studies with integrated TRECs, TL, and TR repertoire diversity assessment in different CD4^+^ and CD8^+^ subsets are needed in order to better understand the role of thymus on immune homeostasis in very long-lasting HIV infection.

## Conclusions

To conclude, our data suggest that in young adults with HIV-infection and on effective cART, thymic function is maintained at least during the first three decades of life, independently of HIV infection lasting. A normal thymic output together with reduced TL and TR repertoire diversity could be explained by the shift of newly produced cells into memory subsets, in order to control viral infection and maintain peripheral homeostasis. However, this phenomenon is more pronounced in pHIVy, which may have a more remarkable thymic reserve, supporting a higher degree of normalization of TR repertoire.

## Supplementary information

**Additional file 1.** Gender-related median values of TRECs and KRECs in perinatally HIV-infected youths (pHIVy), in non-perinatally HIV-infected youths (npHIVy) and in healthy controls (HC).

**Additional file 2.** Correlation between number of (A) TRECs and (B) telomere length (expressed as T/S ratio) with CD4^+^, CD8^+^ cells and CD4/CD8 ratio in perinatally HIV-infected youths (pHIVy), in non-perinatally HIV-infected youths (npHIVy) and in healthy controls (HC).

**Additional file 3.** Relative frequency of individual TRBV subgroup usage in perinatally HIV-infected youths (pHIVy), in non-perinatally HIV-infected youths (npHIVy) and in healthy controls (HC). The relative expression of each TRBV transcript was quantified as described by Gorochov G [50].

**Additional file 4.** Correlation between the percentage of TRBV perturbations and CD4^+^ (A), CD8^+^ lymphocytes (B) and CD4/CD8 ratio (C) in perinatally HIV-infected youths (pHIVy) and in non-perinatally HIV-infected youths (npHIVy).

## Data Availability

The datasets generated and analyzed during the current study are not publicly available due to ethical and law restrictions but are available from the corresponding author on reasonable request.

## Abbreviations

cART: Combined antiretroviral therapy
CDR3: Complementarity determining region 3
HC: Healthy controls
KRECs: K-deleting recombination excision circles
npHIVy: Non perinatally HIV-infected youths
pHIVy: Perinatally HIV-infected youths
qPCR: Quantitative Real-Time PCR
S: Single copy gene
T: Telomere
TL: Telomere length
TR: T-cell receptor
TRBV: TR beta variable
TRECs: TR excision circles
