# Supplementary material for: Lymphocyte homeostasis is maintained in perinatally HIV-infected patients after three decades of life
Source: Immun Ageing. 2019 Oct 13;16:26. doi: 10.1186/s12979-019-0166-7 (PMC6791008; doi:10.1186/s12979-019-0166-7)
Supplement: Supplementary file 2 — Additional file 2. Correlation between number of (A) TRECs and (B) telomere length (expressed as T/S ratio) with CD4+, CD8+ cells and CD4/CD8 ratio in perinatally HIV-infected youths (pHIVy), in non-perinatally HIV-infected youths (npHIVy) and in healthy controls (HC). [file 12979_2019_166_MOESM2_ESM.ppt]

## Slide 1
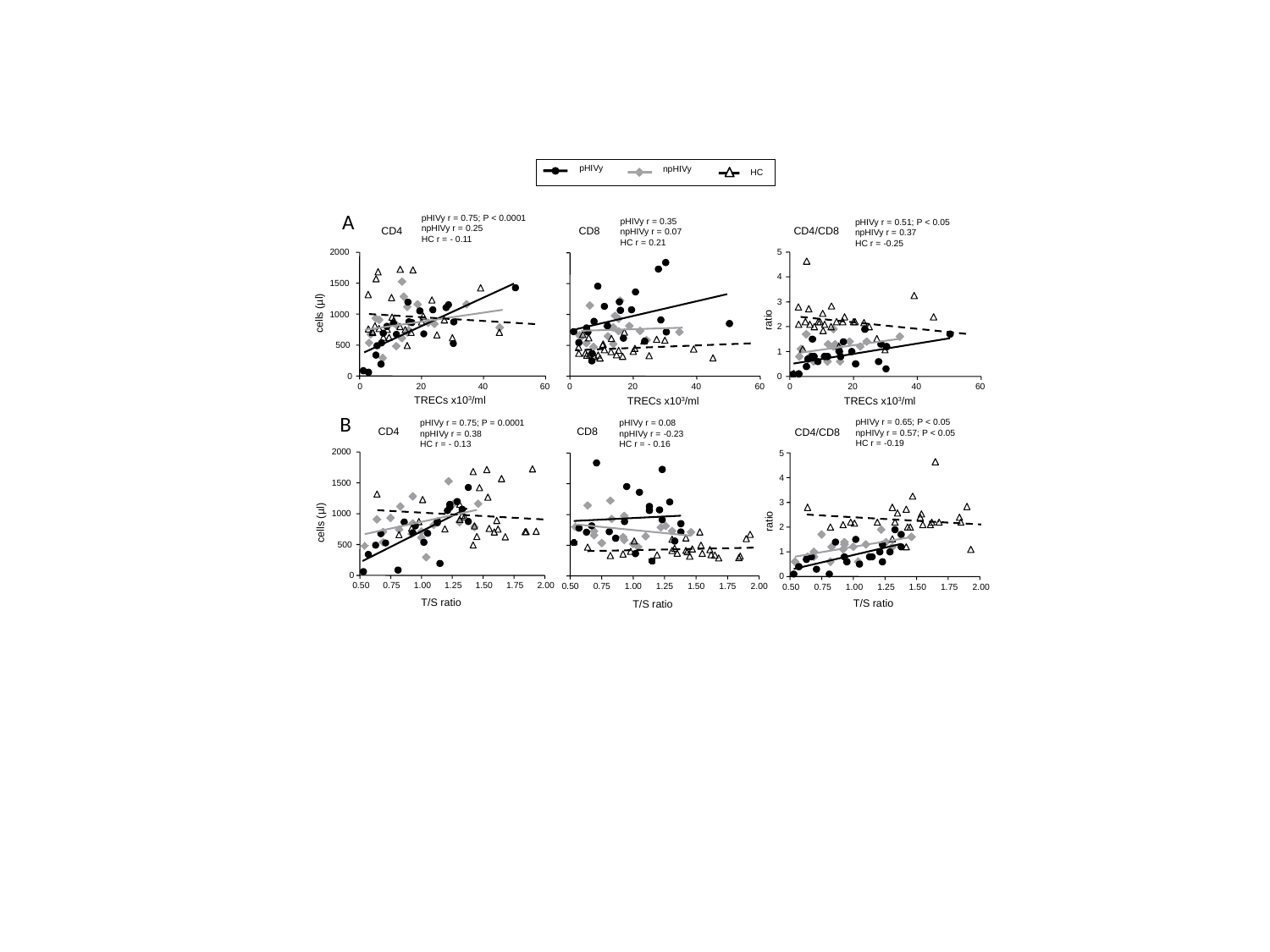

pHIVy
npHIVy
HC
A
pHIVy r = 0.75; P < 0.0001
npHIVy r = 0.25
HC r = - 0.11
pHIVy r = 0.35
npHIVy r = 0.07
HC r = 0.21
pHIVy r = 0.51; P < 0.05
npHIVy r = 0.37
HC r = -0.25
CD8
CD4/CD8
CD4
2000
5
4
1500
ratio
3
cells (µl)
1000
2
500
1
0
0
0
20
40
60
0
20
40
60
0
20
40
60
TRECs x103/ml
TRECs x103/ml
TRECs x103/ml
B
pHIVy r = 0.65; P < 0.05
npHIVy r = 0.57; P < 0.05
HC r = -0.19
pHIVy r = 0.75; P = 0.0001
npHIVy r = 0.38
HC r = - 0.13
pHIVy r = 0.08
npHIVy r = -0.23
HC r = - 0.16
CD4
CD8
CD4/CD8
2000
5
4
1500
ratio
3
1000
cells (µl)
2
500
1
0
0
0.50
0.75
1.00
1.25
1.50
1.75
2.00
0.50
0.75
1.00
1.25
1.50
1.75
2.00
0.50
0.75
1.00
1.25
1.50
1.75
2.00
T/S ratio
T/S ratio
T/S ratio
