# Supplementary figures and images for: Lymphocyte homeostasis is maintained in perinatally HIV-infected patients after three decades of life
Source: Immun Ageing. 2019 Oct 13;16:26. doi: 10.1186/s12979-019-0166-7 (PMC6791008; doi:10.1186/s12979-019-0166-7)

## Slide 1
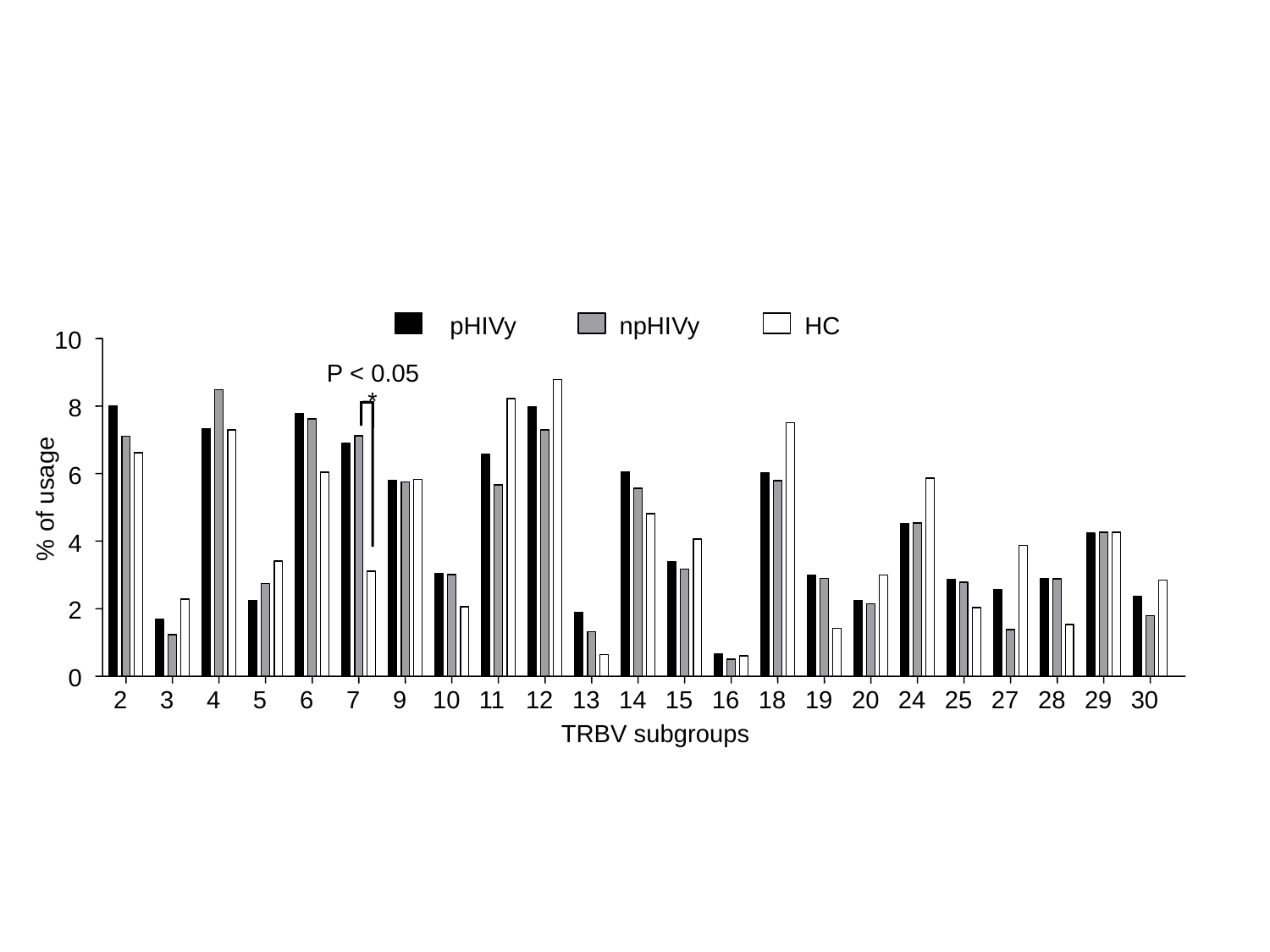

pHIVy
npHIVy
HC
10
P < 0.05
*
8
6
% of usage
4
2
0
2
3
4
5
6
7
9
10
11
12
13
14
15
16
18
19
20
24
25
27
28
29
30
TRBV subgroups

Supplement: Supplementary file 3 — Additional file 3. Relative frequency of individual TRBV subgroup usage in perinatally HIV-infected youths (pHIVy), in non-perinatally HIV-infected youths (npHIVy) and in healthy controls (HC). The relative expression of each TRBV transcript was quantified as described by Gorochov G [50]. [file 12979_2019_166_MOESM3_ESM.ppt]
