# Supplementary material for: Lymphocyte homeostasis is maintained in perinatally HIV-infected patients after three decades of life
Source: Immun Ageing. 2019 Oct 13;16:26. doi: 10.1186/s12979-019-0166-7 (PMC6791008; doi:10.1186/s12979-019-0166-7)
Supplement: Supplementary file 4 — Additional file 4. Correlation between the percentage of TRBV perturbations and CD4+ (A), CD8+ lymphocytes (B) and CD4/CD8 ratio (C) in perinatally HIV-infected youths (pHIVy) and in non-perinatally HIV-infected youths (npHIVy). [file 12979_2019_166_MOESM4_ESM.ppt]

## Slide 1
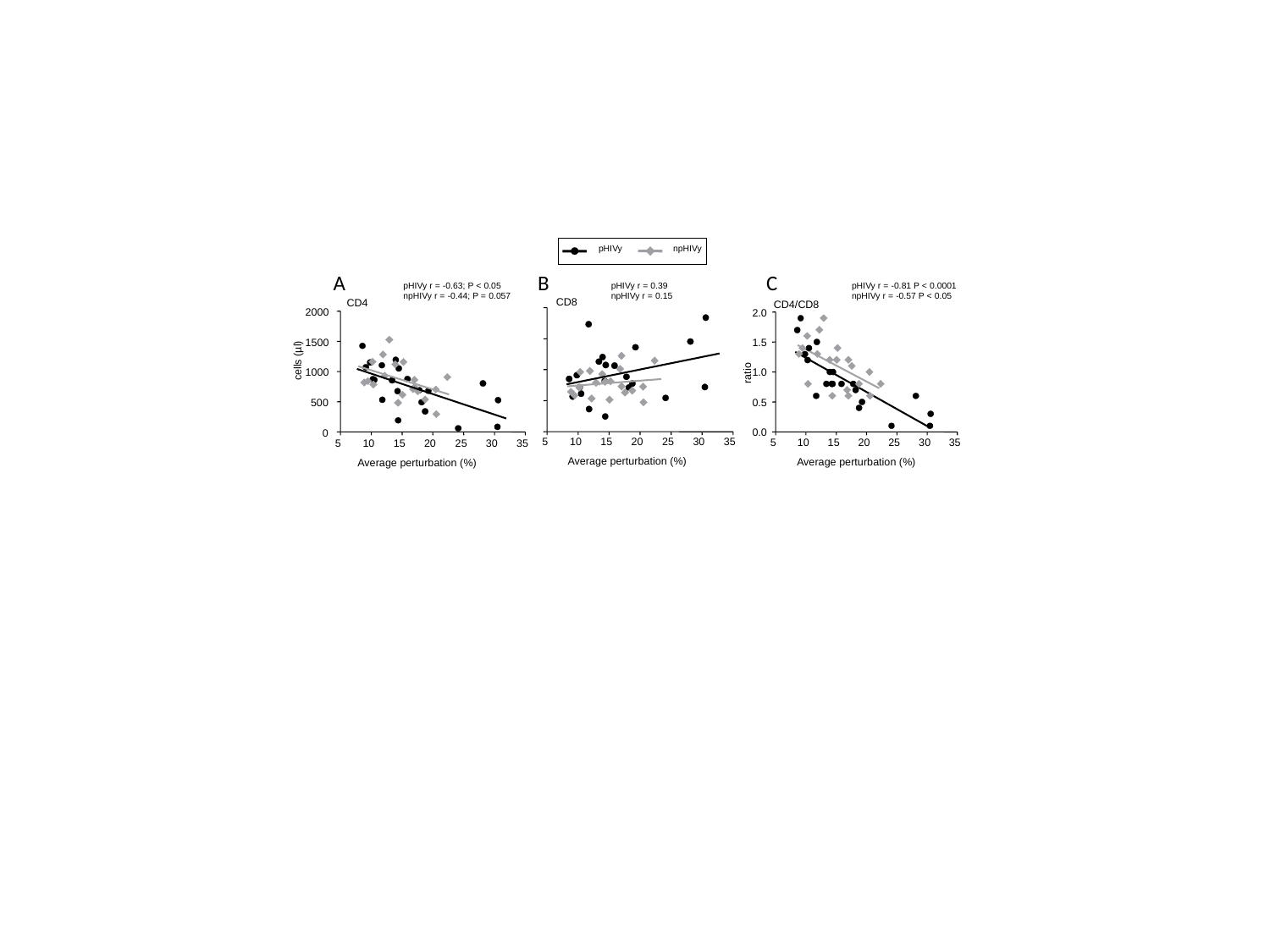

npHIVy
pHIVy
A
B
C
pHIVy r = -0.63; P < 0.05
npHIVy r = -0.44; P = 0.057
pHIVy r = -0.81 P < 0.0001
npHIVy r = -0.57 P < 0.05
pHIVy r = 0.39
npHIVy r = 0.15
CD8
CD4
CD4/CD8
2000
2.0
1500
1.5
ratio
cells (µl)
1000
1.0
0.5
500
0.0
0
5
10
15
20
25
30
35
5
10
15
20
25
30
35
5
10
15
20
25
30
35
Average perturbation (%)
Average perturbation (%)
Average perturbation (%)
